# Supplementary material for: Evidence of Community-Wide Spread of Multi-Drug Resistant Escherichia coli in Young Children in Lusaka and Ndola Districts, Zambia
Source: Microorganisms. 2022 Aug 21;10(8):1684. doi: 10.3390/microorganisms10081684 (PMC9416312; doi:10.3390/microorganisms10081684)
Supplement: Supplementary file 1 [file microorganisms-10-01684-s001.zip › File S1. Questionnaire for guardians.pdf]

Questionnaire serial number: [ ][ ][ ]

Appendix S1 ***Questionnaire- risk factors associated with antimicrobial resistant - E. coli in the underfive year children in Lusaka and Ndola districts***

The purpose of this research study is to gather information on the risk factors associated with antimicrobial resistant - E. coli in the underfive year children. You have been asked to participate in this research study and please note that by completing this questionnaire you are voluntarily agreeing to participate in this study. You will remain anonymous and all information given will be treated as confidential.

Data collectors to mark the appropriate block with an X or write the answer on the space provided.

Section A Demographic Characteristics

For Official Use

1.0 Participant Identification ______________________

2.0 Sex of child

[1] Male [2] Female — [ ] 1

3.0 Date of birth of the child

_____/ _____/ _____
D D M M Y Y — [ ] 2

4.0 Guardian formal education

[0] None (did not attend school)
[1] Primary (grade 1-7)
[2] Junior Secondary (grade 8-9) — [ ] 3
[3] Senior Secondary (grade 10-12)/ Vocational training
[4] Tertiary

5.0 Location of household

[1] Urban [2] Rural — [ ] 4

6.0 Population density in the area of habitation

[1] Low density [2] High density — [ ] 5
[3] Medium density

Housing

7.0 How many people are living in the household? ______ — [ ][ ] 6

18 years and below (Children) ______ — [ ][ ] 7

Above 18 years (Adults) ______ — [ ][ ] 8

8.0 Have you had a visitor in the last 7 day?

[1] Yes [2] No — [ ] 9

9.0 Has someone from the household visited another place in the past 7 days and back?

[1] Yes [2] No — [ ] 10

10.0 What is the household's main source of water for drinking and cooking?

[1] Pipe borne [2] Borehole

[3] River/ Pond/ Dam [4] Sachet/ Bottled/filtered — [ ] 11

[5] Others specify ……………………………………… — [ ][ ] 12-13

11.0 Do you treat or filter drinking water?

[1] Yes [2] No [3] Sometimes — [ ] 14

12.0 Is there any animal kept at your household?

[1] Yes [2] No — [ ] 15

13.0 If yes to question 12, name the types of animal kept at your household — [ ][ ] 16-17 / [ ][ ] 18-19 / [ ][ ] 20-21

____

____

Feeding

14.0 What were you feeding the child during the first 6 months?

[1] Breastmilk only

[2] Breastmilk with other commercial milk/food — [ ] 22

[3] Commercial milk/food only

15.0 Do you wash your hands before preparing or giving food to the child?

[1] Yes [2] No [3] Sometimes — [ ] 23

16.0 Do you wash hands after disposing off the child's faeces?

[1] Yes [2] No [3] Sometimes — [ ] 24

17.0 Where do you normally store the prepared food for the children?

____ — [ ][ ][ ][ ] 25-28

18.0 What do you use to feed the child? *Multiple responses*

[1] Spoon — [ ] 29

[2] Fingers/ Hands — [ ] 30

[3] Others Specify……………………………………………………. — [ ][ ] 31-32

Sanitation

19.0 What toilet facility do you have?

[1] Flush toilet

[2] Pit latrine — [ ] 33

[3] Other (specify)…..

20.0 Do you wash hands after going to the toilet?

1 Yes 2 No 3 Sometimes 34

21.0 How do you dispose off your solid waste?

1 Bin 2 Pit 35

3 Roadside 4 36-37

Knowledge and awareness around antibiotics and diarrhoea

22.0 Do you know antibiotics?

1 Yes 2 No 38

23.0 If yes to question 22, name at least two (2) antibiotics

Antibiotic 1: ____________________

Antibiotic 2: ____________________ 39-41

24.0 Do you know what antibiotic resistance is?

1 Yes 2 No 42

25.0 If yes to question 24, define antibiotic resistance in your own way

____

____________________ 43-45

____

26.0 Is it acceptable to use antibiotics given or suggested by friends or family members as long as they were used to treat the same problem?

1 Yes 2 No 3 Sometimes 46

27.0 Do you know the causes of diarrhoea?

1 Yes 2 No 47

28.0 If yes to question 27, indicate at least two causes of diarrhoea

Cause 1: ____________________

Cause 2: ____________________ 48-49

50-51

Clinical Data

29.0 Date of onset of diarrhoea ____________________

30.0 Duration of diarrhoea

1 …..................... Days

31.0 Number of household members with diarrhoea within 10 days before the patients's illness 52

32.0 Were they treated with antibiotics?

1 Yes 2 No 53

33.0 If yes to question 32, indicate the name of the antibiotics ____________________ ☐☐☐☐ 54-55

34.0 Was there fever at attendance? ☐ 56

☐ 1 Yes ☐ 2 No

35.0 Vomiting during diarrhoea? ☐ 57

☐ 1 Yes ☐ 2 No

36.0 Bloody diarrhoea ☐ 58

☐ 1 Yes ☐ 2 No

37.0 Diarrhoea with mucus ☐ 59

☐ 1 Yes ☐ 2 No

38.0 Date of sample stool collection ____________________ ☐ 60

Thank you for your time and contribution!!!!!
