# Supplementary material for: Evidence of Community-Wide Spread of Multi-Drug Resistant Escherichia coli in Young Children in Lusaka and Ndola Districts, Zambia
Source: Microorganisms. 2022 Aug 21;10(8):1684. doi: 10.3390/microorganisms10081684 (PMC9416312; doi:10.3390/microorganisms10081684)
Supplement: Supplementary file 1 [file microorganisms-10-01684-s001.zip › Table S1. Overall AMR E. coli patterns.pdf]

Supplementary 2. Overall antimicrobial resistant- E. coli patterns
All (N=1020)

Resistance patterns	MDR	XDR	PDR	N* of isolates	%Isolates
				32	3.1
IPM				14	1.4
SXT				9	0.9
TCY				9	0.9
NAL				1	0.1
GEN				2	0.2
CIP				2	0.2
CTX				8	0.8
AMP				9	0.9
AMC				1	0.1
SXT IPM				6	0.6
TCY IPM				2	0.2
TCY SXT				6	0.6
GEN IPM				3	0.3
GEN SXT				1	0.1
CIP IPM				2	0.2
CIP TCY				1	0.1
CIP NAL				2	0.2
CTX IPM				6	0.6
CTX SXT				1	0.1
CTX TCY				1	0.1
CTX NAL				1	0.1
AMP IPM				9	0.9
AMP SXT				19	1.9
AMP TCY				7	0.7
AMP CIP				1	0.1
AMP CHL				2	0.2
AMP CTX				8	0.8
AMC IPM				3	0.3
AMC CIP				1	0.1
CTX CIP NAL				5	0.5
AMC CIP NAL				1	0.1
CHL NAL TCY	MDR			1	0.1
CTX NAL IPM	MDR			1	0.1
CTX NAL TCY	MDR			2	0.2
CTX GEN NAL	MDR			1	0.1
AMP SXT IPM	MDR			16	1.6
AMP TCY IPM	MDR			6	0.6
AMP TCY SXT	MDR			76	7.5
AMP NAL SXT	MDR			3	0.3
AMP NAL TCY	MDR			2	0.2
AMP GEN SXT	MDR			2	0.2
AMP CIP IPM	MDR			2	0.2
AMP CIP SXT	MDR			1	0.1
AMP CHL SXT	MDR			2	0.2
AMP CTX IPM	MDR			1	0.1

AMP CTX SXT	MDR	9	0.9
AMP CTX TCY	MDR	3	0.3
AMP CTX NAL	MDR	3	0.3
AMP CTX CIP	MDR	1	0.1
AMC AMP IPM	MDR	1	0.1
AMC AMP SXT	MDR	6	0.6
AMC AMP TCY	MDR	4	0.4
AMC AMP CTX	MDR	15	1.5
AMP NAL SXT IPM	MDR	1	0.1
AMP NAL TCY SXT	MDR	7	0.7
AMP CIP NAL SXT	MDR	2	0.2
AMP CIP NAL TCY	MDR	2	0.2
AMP CHL NAL TCY	MDR	2	0.2
AMP CTX NAL IPM	MDR	1	0.1
AMP CTX NAL SXT	MDR	3	0.3
AMP CTX NAL TCY	MDR	1	0.1
AMP CTX CIP NAL	MDR	2	0.2
AMC AMP NAL SXT	MDR	1	0.1
AMC AMP NAL TCY	MDR	1	0.1
TCY SXT IPM	MDR	6	0.6
CIP TCY IPM	MDR	2	0.2
CIP TCY SXT	MDR	1	0.1
CTX SXT IPM	MDR	1	0.1
CTX CIP IPM	MDR	1	0.1
CTX CIP SXT	MDR	1	0.1
CTX CIP TCY	MDR	1	0.1
CTX CHL IPM	MDR	1	0.1
AMC CTX CIP	MDR	2	0.2
GEN TCY SXT IPM	MDR	1	0.1
CIP TCY SXT IPM	MDR	2	0.2
CIP NAL SXT IPM	MDR	1	0.1
CIP NAL TCY IPM	MDR	1	0.1
CIP NAL TCY SXT	MDR	1	0.1
CIP GEN SXT IPM	MDR	1	0.1
CIP GEN TCY IPM	MDR	1	0.1
CTX TCY SXT IPM	MDR	1	0.1
CTX NAL TCY SXT	MDR	3	0.3
CTX CIP NAL IPM	MDR	2	0.2
CTX CIP NAL TCY	MDR	1	0.1
AMP TCY SXT IPM	MDR	30	2.9
AMP GEN TCY SXT	MDR	2	0.2
AMP CIP SXT IPM	MDR	2	0.2
AMP CIP TCY SXT	MDR	10	1.0
AMP CHL SXT IPM	MDR	4	0.4
AMP CHL TCY IPM	MDR	1	0.1
AMP CHL TCY SXT	MDR	4	0.4
AMP CTX SXT IPM	MDR	3	0.3
AMP CTX TCY IPM	MDR	1	0.1
AMP CTX TCY SXT	MDR	35	3.4
AMP CTX GEN IPM	MDR	1	0.1

AMP CTX GEN TCY	MDR	2	0.2
AMP CTX CIP IPM	MDR	4	0.4
AMP CTX CIP TCY	MDR	3	0.3
AMP CTX CIP GEN	MDR	2	0.2
AMP CTX CHL SXT	MDR	2	0.2
AMC CTX SXT IPM	MDR	1	0.1
AMC AMP SXT IPM	MDR	5	0.5
AMC AMP TCY IPM	MDR	1	0.1
AMC AMP TCY SXT	MDR	16	1.6
AMC AMP GEN TCY	MDR	1	0.1
AMC AMP CIP SXT	MDR	3	0.3
AMC AMP CHL SXT	MDR	2	0.2
AMC AMP CTX IPM	MDR	3	0.3
AMC AMP CTX SXT	MDR	11	1.1
AMC AMP CTX TCY	MDR	4	0.4
AMC AMP CTX CIP	MDR	4	0.4
CIP NAL TCY SXT IPM	MDR	2	0.2
CIP GEN NAL TCY SXT	MDR	1	0.1
CTX NAL TCY SXT IPM	MDR	1	0.1
CTX GEN TCY SXT IPM	MDR	1	0.1
CTX CIP GEN SXT IPM	MDR	2	0.2
CTX CIP GEN TCY IPM	MDR	1	0.1
CTX CIP GEN NAL TCY	MDR	1	0.1
CTX CHL TCY SXT IPM	MDR	1	0.1
CTX CHL CIP NAL IPM	MDR	1	0.1
AMP NAL TCY SXT IPM	MDR	2	0.2
AMP GEN TCY SXT IPM	MDR	5	0.5
AMP GEN NAL TCY SXT	MDR	2	0.2
AMP CIP TCY SXT IPM	MDR	3	0.3
AMP CIP NAL TCY IPM	MDR	1	0.1
AMP CIP NAL TCY SXT	MDR	6	0.6
AMP CHL TCY SXT IPM	MDR	6	0.6
AMP CHL NAL TCY IPM	MDR	1	0.1
AMP CHL NAL TCY SXT	MDR	5	0.5
AMP CHL CIP SXT IPM	MDR	2	0.2
AMP CHL CIP TCY SXT	MDR	1	0.1
AMP CTX TCY SXT IPM	MDR	4	0.4
AMP CTX NAL SXT IPM	MDR	2	0.2
AMP CTX NAL TCY SXT	MDR	10	1.0
AMP CTX GEN TCY SXT	MDR	1	0.1
AMP CTX GEN NAL TCY	MDR	1	0.1
AMP CTX CIP NAL SXT	MDR	2	0.2
AMP CTX CIP GEN SXT	MDR	1	0.1
AMP CTX CIP GEN NAL	MDR	1	0.1
AMP CTX CHL TCY SXT	MDR	3	0.3
AMP CTX CHL NAL SXT	MDR	2	0.2
AMP CTX CHL NAL TCY	MDR	1	0.1
AMC CTX TCY SXT IPM	MDR	1	0.1
AMC CTX GEN SXT IPM	MDR	1	0.1
AMC CTX CIP TCY IPM	MDR	1	0.1

AMC AMP TCY SXT IPM	MDR	14	1.4
AMC AMP NAL TCY SXT	MDR	7	0.7
AMC AMP GEN SXT IPM	MDR	1	0.1
AMC AMP CIP SXT IPM	MDR	1	0.1
AMC AMP CIP TCY SXT	MDR	3	0.3
AMC AMP CIP NAL TCY	MDR	1	0.1
AMC AMP CHL SXT IPM	MDR	1	0.1
AMC AMP CHL TCY SXT	MDR	4	0.4
AMC AMP CHL NAL SXT	MDR	1	0.1
AMC AMP CHL CIP SXT	MDR	1	0.1
AMC AMP CTX SXT IPM	MDR	9	0.9
AMC AMP CTX TCY IPM	MDR	1	0.1
AMC AMP CTX TCY SXT	MDR	21	2.1
AMC AMP CTX NAL IPM	MDR	2	0.2
AMC AMP CTX NAL SXT	MDR	2	0.2
AMC AMP CTX GEN IPM	MDR	1	0.1
AMC AMP CTX CIP IPM	MDR	3	0.3
AMC AMP CTX CIP SXT	MDR	1	0.1
AMC AMP CTX CIP NAL	MDR	1	0.1
AMC AMP CTX CHL SXT	MDR	1	0.1
CTX CIP GEN NAL SXT IPM	MDR	1	0.1
CTX CIP GEN NAL TCY IPM	MDR	1	0.1
CTX CIP GEN NAL TCY SXT	MDR	1	0.1
AMP CIP NAL TCY SXT IPM	MDR	6	0.6
AMP CIP GEN TCY SXT IPM	MDR	2	0.2
AMP CIP GEN NAL TCY SXT	MDR	5	0.5
AMP CHL NAL TCY SXT IPM	MDR	1	0.1
AMP CHL GEN TCY SXT IPM	MDR	1	0.1
AMP CHL GEN NAL SXT IPM	MDR	1	0.1
AMP CHL CIP NAL TCY SXT	MDR	2	0.2
AMP CTX NAL TCY SXT IPM	MDR	3	0.3
AMP CTX GEN NAL SXT IPM	MDR	1	0.1
AMP CTX CIP NAL TCY IPM	MDR	1	0.1
AMP CTX CIP NAL TCY SXT	MDR	8	0.8
AMP CTX CIP GEN TCY IPM	MDR	1	0.1
AMP CTX CIP GEN TCY SXT	MDR	1	0.1
AMP CTX CIP GEN NAL IPM	MDR	1	0.1
AMP CTX CIP GEN NAL SXT	MDR	1	0.1
AMP CTX CHL NAL TCY SXT	MDR	1	0.1
AMP CTX CHL GEN NAL IPM	MDR	1	0.1
AMP CTX CHL CIP TCY SXT	MDR	1	0.1
AMP CTX CHL CIP GEN SXT	MDR	1	0.1
AMC CHL CIP NAL TCY SXT	MDR	1	0.1
AMC AMP GEN TCY SXT IPM	MDR	2	0.2
AMC AMP GEN NAL SXT IPM	MDR	1	0.1
AMC AMP CIP TCY SXT IPM	MDR	1	0.1
AMC AMP CIP GEN NAL SXT	MDR	2	0.2
AMC AMP CHL TCY SXT IPM	MDR	3	0.3
AMC AMP CHL NAL TCY SXT	MDR	1	0.1
AMC AMP CHL GEN TCY SXT	MDR	2	0.2

AMC AMP CHL CIP TCY SXT	MDR		2	0.2
AMC AMP CHL CIP NAL SXT	MDR		1	0.1
AMC AMP CTX TCY SXT IPM	MDR		13	1.3
AMC AMP CTX NAL SXT IPM	MDR		2	0.2
AMC AMP CTX NAL TCY SXT	MDR		3	0.3
AMC AMP CTX GEN SXT IPM	MDR		3	0.3
AMC AMP CTX GEN TCY IPM	MDR		2	0.2
AMC AMP CTX GEN TCY SXT	MDR		2	0.2
AMC AMP CTX CIP SXT IPM	MDR		1	0.1
AMC AMP CTX CIP TCY IPM	MDR		2	0.2
AMC AMP CTX CIP TCY SXT	MDR		6	0.6
AMC AMP CTX CIP NAL IPM	MDR		4	0.4
AMC AMP CTX CIP NAL TCY	MDR		1	0.1
AMC AMP CTX CIP GEN IPM	MDR		1	0.1
AMC AMP CTX CIP GEN TCY	MDR		1	0.1
AMC AMP CTX CIP GEN NAL	MDR		2	0.2
AMC AMP CTX CHL TCY IPM	MDR		1	0.1
AMC AMP CTX CHL TCY SXT	MDR		6	0.6
AMC AMP CTX CHL CIP NAL	MDR		2	0.2
AMP CIP GEN NAL TCY SXT IPM	MDR		1	0.1
AMP CHL GEN NAL TCY SXT IPM	MDR		1	0.1
AMP CHL CIP NAL TCY SXT IPM	MDR		2	0.2
AMP CTX GEN NAL TCY SXT IPM	MDR		1	0.1
AMP CTX CIP NAL TCY SXT IPM	MDR		5	0.5
AMP CTX CIP GEN NAL TCY IPM	MDR		4	0.4
AMP CTX CIP GEN NAL TCY SXT	MDR		10	1.0
AMP CTX CHL NAL TCY SXT IPM	MDR		2	0.2
AMP CTX CHL GEN NAL TCY SXT	MDR		1	0.1
AMP CTX CHL CIP NAL TCY SXT	MDR		3	0.3
AMP CTX CHL CIP GEN NAL IPM	MDR		1	0.1
AMC AMP CIP NAL TCY SXT IPM	MDR		1	0.1
AMC AMP CIP GEN NAL TCY SXT	MDR		1	0.1
AMC AMP CHL NAL TCY SXT IPM	MDR		4	0.4
AMC AMP CHL CIP NAL TCY SXT	MDR		1	0.1
AMC AMP CTX NAL TCY SXT IPM	MDR		4	0.4
AMC AMP CTX GEN NAL TCY IPM	MDR		1	0.1
AMC AMP CTX GEN NAL TCY SXT	MDR		4	0.4
AMC AMP CTX CIP NAL SXT IPM	MDR		1	0.1
AMC AMP CTX CIP NAL TCY IPM	MDR		2	0.2
AMC AMP CTX CIP NAL TCY SXT	MDR		13	1.3
AMC AMP CTX CIP GEN NAL SXT	MDR		1	0.1
AMC AMP CTX CIP GEN NAL TCY	MDR		2	0.2
AMC AMP CTX CHL NAL TCY SXT	MDR		2	0.2
AMC AMP CTX CHL CIP NAL SXT	MDR		1	0.1
CTX CHL GEN TCY SXT IPM	MDR	Possible XDR	1	0.1
AMC CTX GEN TCY SXT IPM	MDR	Possible XDR	1	0.1
CTX CIP GEN NAL TCY SXT IPM	MDR	Possible XDR	1	0.1
CTX CHL CIP GEN NAL TCY IPM	MDR	Possible XDR	1	0.1
AMP CHL CIP GEN TCY SXT IPM	MDR	Possible XDR	1	0.1
AMP CTX CIP GEN TCY SXT IPM	MDR	Possible XDR	2	0.2

AMP CTX CHL CIP GEN TCY SXT	MDR	Possible XDR		1	0.1
AMC AMP CHL GEN TCY SXT IPM	MDR	Possible XDR		1	0.1
AMC AMP CHL CIP TCY SXT IPM	MDR	Possible XDR		1	0.1
AMC AMP CTX GEN TCY SXT IPM	MDR	Possible XDR		2	0.2
AMC AMP CTX CIP TCY SXT IPM	MDR	Possible XDR		8	0.8
AMC AMP CTX CIP GEN TCY IPM	MDR	Possible XDR		1	0.1
AMC AMP CTX CIP GEN TCY SXT	MDR	Possible XDR		1	0.1
AMC AMP CTX CHL TCY SXT IPM	MDR	Possible XDR		3	0.3
AMC AMP CTX CHL GEN TCY SXT	MDR	Possible XDR		1	0.1
AMC AMP CTX CHL CIP TCY SXT	MDR	Possible XDR		1	0.1
AMP CTX CIP GEN NAL TCY SXT IPM	MDR	Possible XDR		6	0.6
AMP CTX CHL CIP NAL TCY SXT IPM	MDR	Possible XDR		1	0.1
AMP CTX CHL CIP GEN NAL TCY SXT	MDR	Possible XDR		2	0.2
AMC AMP CIP GEN NAL TCY SXT IPM	MDR	Possible XDR		2	0.2
AMC AMP CHL CIP NAL TCY SXT IPM	MDR	Possible XDR		1	0.1
AMC AMP CTX GEN NAL TCY SXT IPM	MDR	Possible XDR		5	0.5
AMC AMP CTX CIP NAL TCY SXT IPM	MDR	Possible XDR		15	1.5
AMC AMP CTX CIP GEN TCY SXT IPM	MDR	Possible XDR		4	0.4
AMC AMP CTX CIP GEN NAL SXT IPM	MDR	Possible XDR		5	0.5
AMC AMP CTX CIP GEN NAL TCY SXT	MDR	Possible XDR		12	1.2
AMC AMP CTX CHL NAL TCY SXT IPM	MDR	Possible XDR		6	0.6
AMC AMP CTX CHL GEN TCY SXT IPM	MDR	Possible XDR		1	0.1
AMC AMP CTX CHL CIP TCY SXT IPM	MDR	Possible XDR		3	0.3
AMC AMP CTX CHL CIP NAL SXT IPM	MDR	Possible XDR		3	0.3
AMC AMP CTX CHL CIP NAL TCY IPM	MDR	Possible XDR		1	0.1
AMC AMP CTX CHL CIP NAL TCY SXT	MDR	Possible XDR		2	0.2
AMC AMP CTX CHL CIP GEN TCY SXT	MDR	Possible XDR		2	0.2
AMC AMP CTX CHL CIP GEN NAL SXT	MDR	Possible XDR		1	0.1
AMC AMP CTX CHL CIP GEN NAL TCY	MDR	Possible XDR		1	0.1
AMP CTX CHL CIP GEN NAL TCY SXT IPM	MDR	Possible XDR		3	0.3
AMC AMP CTX CIP GEN NAL TCY SXT IPM	MDR	Possible XDR		13	1.3
AMC AMP CTX CHL GEN NAL TCY SXT IPM	MDR	Possible XDR		1	0.1
AMC AMP CTX CHL CIP NAL TCY SXT IPM	MDR	Possible XDR		2	0.2
AMC AMP CTX CHL CIP GEN NAL SXT IPM	MDR	Possible XDR		2	0.2
AMC AMP CTX CHL CIP GEN NAL TCY SXT	MDR	Possible XDR		15	1.5
AMC AMP CTX CHL CIP GEN TCY SXT IPM	MDR	Possible XDR	Possible PDR	3	0.3
AMC AMP CTX CHL CIP GEN NAL TCY SXT IPM	MDR	Possible XDR	Possible PDR	6	0.6
