# Supplementary material for: Evidence of Community-Wide Spread of Multi-Drug Resistant Escherichia coli in Young Children in Lusaka and Ndola Districts, Zambia
Source: Microorganisms. 2022 Aug 21;10(8):1684. doi: 10.3390/microorganisms10081684 (PMC9416312; doi:10.3390/microorganisms10081684)
Supplement: Supplementary file 1 [file microorganisms-10-01684-s001.zip › Table S2. Healthy Children AMR E. coli patterns.pdf]

Supplementary 3. Antimicrobial resistant- E. coli patterns in healthy children

Healthy (N=455)

Resistance patterns	MDR	XDR	PDR	N* of isolates	%Isolates
				21	4.6
IPM				9	2.0
SXT				1	0.2
TCY				4	0.9
GEN				2	0.4
CIP				1	0.2
CTX				4	0.9
SXT IPM				6	1.3
TCY IPM				2	0.4
TCY SXT				3	0.7
GEN IPM				3	0.7
CIP IPM				1	0.2
CIP NAL				1	0.2
CTX IPM				4	0.9
CTX NAL				1	0.2
AMP IPM				6	1.3
AMP SXT				4	0.9
AMP TCY				1	0.2
AMP CTX				2	0.4
AMC IPM				3	0.7
CTX CIP NAL				1	0.2
CTX GEN NAL	MDR			1	0.2
AMP SXT IPM	MDR			13	2.9
AMP TCY IPM	MDR			4	0.9
AMP TCY SXT	MDR			17	3.7
AMP NAL TCY	MDR			1	0.2
AMP GEN SXT	MDR			1	0.2
AMP CIP IPM	MDR			2	0.4
AMP CIP SXT	MDR			1	0.2
AMP CTX IPM	MDR			1	0.2
AMP CTX SXT	MDR			1	0.2
AMP CTX CIP	MDR			1	0.2
AMC AMP IPM	MDR			1	0.2
AMC AMP SXT	MDR			3	0.7
AMC AMP TCY	MDR			3	0.7
AMC AMP CTX	MDR			5	1.1
AMP NAL SXT IPM	MDR			1	0.2
AMP NAL TCY SXT	MDR			1	0.2
AMP CIP NAL SXT	MDR			1	0.2
AMP CHL NAL TCY	MDR			1	0.2
AMP CTX NAL IPM	MDR			1	0.2
AMP CTX NAL SXT	MDR			2	0.4
AMP CTX NAL TCY	MDR			1	0.2
AMC AMP NAL SXT	MDR			1	0.2
TCY SXT IPM	MDR			3	0.7
CIP TCY IPM	MDR			1	0.2

CIP TCY SXT	MDR	1	0.2
CTX SXT IPM	MDR	1	0.2
CTX CIP IPM	MDR	1	0.2
CTX CIP SXT	MDR	1	0.2
CTX CIP TCY	MDR	1	0.2
CTX CHL IPM	MDR	1	0.2
GEN TCY SXT IPM	MDR	1	0.2
CIP TCY SXT IPM	MDR	1	0.2
CIP NAL SXT IPM	MDR	1	0.2
CIP GEN SXT IPM	MDR	1	0.2
CTX TCY SXT IPM	MDR	1	0.2
CTX NAL TCY SXT	MDR	1	0.2
CTX CIP NAL TCY	MDR	1	0.2
AMP TCY SXT IPM	MDR	17	3.7
AMP GEN TCY SXT	MDR	1	0.2
AMP CIP SXT IPM	MDR	1	0.2
AMP CIP TCY SXT	MDR	3	0.7
AMP CHL SXT IPM	MDR	2	0.4
AMP CHL TCY SXT	MDR	1	0.2
AMP CTX SXT IPM	MDR	1	0.2
AMP CTX TCY SXT	MDR	2	0.4
AMP CTX GEN TCY	MDR	1	0.2
AMP CTX CIP IPM	MDR	4	0.9
AMP CTX CIP TCY	MDR	1	0.2
AMP CTX CIP GEN	MDR	1	0.2
AMP CTX CHL SXT	MDR	1	0.2
AMC AMP SXT IPM	MDR	4	0.9
AMC AMP TCY SXT	MDR	6	1.3
AMC AMP CIP SXT	MDR	1	0.2
AMC AMP CHL SXT	MDR	1	0.2
AMC AMP CTX IPM	MDR	2	0.4
AMC AMP CTX SXT	MDR	5	1.1
AMC AMP CTX TCY	MDR	2	0.4
AMC AMP CTX CIP	MDR	2	0.4
CIP NAL TCY SXT IPM	MDR	1	0.2
CIP GEN NAL TCY SXT	MDR	1	0.2
CTX CIP GEN SXT IPM	MDR	1	0.2
CTX CIP GEN TCY IPM	MDR	1	0.2
CTX CHL TCY SXT IPM	MDR	1	0.2
AMP GEN TCY SXT IPM	MDR	2	0.4
AMP CIP TCY SXT IPM	MDR	2	0.4
AMP CIP NAL TCY SXT	MDR	2	0.4
AMP CHL TCY SXT IPM	MDR	6	1.3
AMP CHL NAL TCY IPM	MDR	1	0.2
AMP CHL CIP SXT IPM	MDR	2	0.4
AMP CHL CIP TCY SXT	MDR	1	0.2
AMP CTX TCY SXT IPM	MDR	2	0.4
AMP CTX NAL SXT IPM	MDR	1	0.2
AMP CTX GEN TCY SXT	MDR	1	0.2
AMP CTX GEN NAL TCY	MDR	1	0.2

AMP CTX CIP NAL SXT	MDR	2	0.4
AMC CTX TCY SXT IPM	MDR	1	0.2
AMC CTX GEN SXT IPM	MDR	1	0.2
AMC AMP TCY SXT IPM	MDR	11	2.4
AMC AMP GEN SXT IPM	MDR	1	0.2
AMC AMP CIP SXT IPM	MDR	1	0.2
AMC AMP CHL SXT IPM	MDR	1	0.2
AMC AMP CHL CIP SXT	MDR	1	0.2
AMC AMP CTX SXT IPM	MDR	5	1.1
AMC AMP CTX TCY IPM	MDR	1	0.2
AMC AMP CTX TCY SXT	MDR	4	0.9
AMC AMP CTX NAL IPM	MDR	1	0.2
AMC AMP CTX GEN IPM	MDR	1	0.2
AMC AMP CTX CIP IPM	MDR	3	0.7
AMC AMP CTX CHL SXT	MDR	1	0.2
CTX CIP GEN NAL SXT IPM	MDR	1	0.2
AMP CIP NAL TCY SXT IPM	MDR	5	1.1
AMP CIP GEN TCY SXT IPM	MDR	2	0.4
AMP CIP GEN NAL TCY SXT	MDR	1	0.2
AMP CHL NAL TCY SXT IPM	MDR	1	0.2
AMP CHL GEN TCY SXT IPM	MDR	1	0.2
AMP CHL CIP NAL TCY SXT	MDR	1	0.2
AMP CTX NAL TCY SXT IPM	MDR	1	0.2
AMP CTX CIP NAL TCY SXT	MDR	3	0.7
AMP CTX CIP GEN TCY IPM	MDR	1	0.2
AMP CTX CIP GEN TCY SXT	MDR	1	0.2
AMP CTX CIP GEN NAL IPM	MDR	1	0.2
AMP CTX CIP GEN NAL SXT	MDR	1	0.2
AMP CTX CHL NAL TCY SXT	MDR	1	0.2
AMP CTX CHL CIP GEN SXT	MDR	1	0.2
AMC CHL CIP NAL TCY SXT	MDR	1	0.2
AMC AMP GEN TCY SXT IPM	MDR	2	0.4
AMC AMP GEN NAL SXT IPM	MDR	1	0.2
AMC AMP CIP TCY SXT IPM	MDR	1	0.2
AMC AMP CHL TCY SXT IPM	MDR	2	0.4
AMC AMP CHL GEN TCY SXT	MDR	2	0.4
AMC AMP CTX TCY SXT IPM	MDR	9	2.0
AMC AMP CTX NAL SXT IPM	MDR	1	0.2
AMC AMP CTX GEN SXT IPM	MDR	2	0.4
AMC AMP CTX GEN TCY IPM	MDR	2	0.4
AMC AMP CTX GEN TCY SXT	MDR	1	0.2
AMC AMP CTX CIP SXT IPM	MDR	1	0.2
AMC AMP CTX CIP TCY IPM	MDR	2	0.4
AMC AMP CTX CIP TCY SXT	MDR	1	0.2
AMC AMP CTX CIP NAL IPM	MDR	3	0.7
AMC AMP CTX CIP GEN IPM	MDR	1	0.2
AMC AMP CTX CIP GEN NAL	MDR	1	0.2
AMC AMP CTX CHL TCY SXT	MDR	1	0.2
AMP CIP GEN NAL TCY SXT IPM	MDR	1	0.2
AMP CHL GEN NAL TCY SXT IPM	MDR	1	0.2

AMP CHL CIP NAL TCY SXT IPM	MDR			2	0.4
AMP CTX GEN NAL TCY SXT IPM	MDR			1	0.2
AMP CTX CIP NAL TCY SXT IPM	MDR			2	0.4
AMP CTX CIP GEN NAL TCY IPM	MDR			4	0.9
AMP CTX CIP GEN NAL TCY SXT	MDR			5	1.1
AMP CTX CHL CIP GEN NAL IPM	MDR			1	0.2
AMC AMP CHL NAL TCY SXT IPM	MDR			1	0.2
AMC AMP CTX NAL TCY SXT IPM	MDR			2	0.4
AMC AMP CTX GEN NAL TCY SXT	MDR			2	0.4
AMC AMP CTX CIP NAL TCY IPM	MDR			2	0.4
AMC AMP CTX CIP NAL TCY SXT	MDR			6	1.3
AMC AMP CTX CIP GEN NAL SXT	MDR			1	0.2
AMC AMP CTX CIP GEN NAL TCY	MDR			2	0.4
CTX CHL GEN TCY SXT IPM	MDR	Possible XDR		1	0.2
AMC CTX GEN TCY SXT IPM	MDR	Possible XDR		1	0.2
AMP CTX CIP GEN TCY SXT IPM	MDR	Possible XDR		1	0.2
AMC AMP CHL GEN TCY SXT IPM	MDR	Possible XDR		1	0.2
AMC AMP CTX GEN TCY SXT IPM	MDR	Possible XDR		2	0.4
AMC AMP CTX CIP TCY SXT IPM	MDR	Possible XDR		7	1.5
AMC AMP CTX CIP GEN TCY IPM	MDR	Possible XDR		1	0.2
AMC AMP CTX CHL TCY SXT IPM	MDR	Possible XDR		3	0.7
AMP CTX CIP GEN NAL TCY SXT IPM	MDR	Possible XDR		5	1.1
AMP CTX CHL CIP NAL TCY SXT IPM	MDR	Possible XDR		1	0.2
AMP CTX CHL CIP GEN NAL TCY SXT	MDR	Possible XDR		2	0.4
AMC AMP CIP GEN NAL TCY SXT IPM	MDR	Possible XDR		1	0.2
AMC AMP CTX GEN NAL TCY SXT IPM	MDR	Possible XDR		4	0.9
AMC AMP CTX CIP NAL TCY SXT IPM	MDR	Possible XDR		9	2.0
AMC AMP CTX CIP GEN TCY SXT IPM	MDR	Possible XDR		2	0.4
AMC AMP CTX CIP GEN NAL SXT IPM	MDR	Possible XDR		4	0.9
AMC AMP CTX CIP GEN NAL TCY SXT	MDR	Possible XDR		3	0.7
AMC AMP CTX CHL NAL TCY SXT IPM	MDR	Possible XDR		4	0.9
AMC AMP CTX CHL GEN TCY SXT IPM	MDR	Possible XDR		1	0.2
AMC AMP CTX CHL CIP TCY SXT IPM	MDR	Possible XDR		2	0.4
AMC AMP CTX CHL CIP NAL SXT IPM	MDR	Possible XDR		2	0.4
AMP CTX CHL CIP GEN NAL TCY SXT IPM	MDR	Possible XDR		2	0.4
AMC AMP CTX CIP GEN NAL TCY SXT IPM	MDR	Possible XDR		10	2.2
AMC AMP CTX CHL GEN NAL TCY SXT IPM	MDR	Possible XDR		1	0.2
AMC AMP CTX CHL CIP NAL TCY SXT IPM	MDR	Possible XDR		1	0.2
AMC AMP CTX CHL CIP GEN NAL SXT IPM	MDR	Possible XDR		1	0.2
AMC AMP CTX CHL CIP GEN NAL TCY SXT	MDR	Possible XDR		4	0.9
AMC AMP CTX CHL CIP GEN TCY SXT IPM	MDR	Possible XDR	Possible PDR	3	0.7
AMC AMP CTX CHL CIP GEN NAL TCY SXT IPM	MDR	Possible XDR	Possible PDR	6	1.3
