# Supplementary material for: Evidence of Community-Wide Spread of Multi-Drug Resistant Escherichia coli in Young Children in Lusaka and Ndola Districts, Zambia
Source: Microorganisms. 2022 Aug 21;10(8):1684. doi: 10.3390/microorganisms10081684 (PMC9416312; doi:10.3390/microorganisms10081684)
Supplement: Supplementary file 1 [file microorganisms-10-01684-s001.zip › Table S3. Diarrhoeic Children AMR E. coli patterns.pdf]

Supplementary 4. Antimicrobial resistant- E. coli patterns in diarrhoeic children
Diarrhoea (N= 565)

Resistance patterns	MDR	XDR	PDR	N* of isolates	%Isolates
				11	1.9
IPM				5	0.9
SXT				8	1.4
TCY				5	0.9
NAL				1	0.2
CIP				1	0.2
CTX				4	0.7
AMP				9	1.6
AMC				1	0.2
TCY SXT				3	0.5
GEN SXT				1	0.2
CIP IPM				1	0.2
CIP TCY				1	0.2
CIP NAL				1	0.2
CTX IPM				2	0.4
CTX SXT				1	0.2
CTX TCY				1	0.2
AMP IPM				3	0.5
AMP SXT				15	2.7
AMP TCY				6	1.1
AMP CIP				1	0.2
AMP CHL				2	0.4
AMP CTX				6	1.1
AMC CIP				1	0.2
CTX CIP NAL				4	0.7
AMC CIP NAL				1	0.2
CHL NAL TCY	MDR			1	0.2
CTX NAL IPM	MDR			1	0.2
CTX NAL TCY	MDR			2	0.4
AMP SXT IPM	MDR			3	0.5
AMP TCY IPM	MDR			2	0.4
AMP TCY SXT	MDR			59	10.4
AMP NAL SXT	MDR			3	0.5
AMP NAL TCY	MDR			1	0.2
AMP GEN SXT	MDR			1	0.2
AMP CHL SXT	MDR			2	0.4
AMP CTX SXT	MDR			8	1.4
AMP CTX TCY	MDR			3	0.5
AMP CTX NAL	MDR			3	0.5
AMC AMP SXT	MDR			3	0.5
AMC AMP TCY	MDR			1	0.2
AMC AMP CTX	MDR			10	1.8
AMP NAL TCY SXT	MDR			6	1.1

AMP CIP NAL SXT	MDR	1	0.2
AMP CIP NAL TCY	MDR	2	0.4
AMP CHL NAL TCY	MDR	1	0.2
AMP CTX NAL SXT	MDR	1	0.2
AMP CTX CIP NAL	MDR	2	0.4
AMC AMP NAL TCY	MDR	1	0.2
TCY SXT IPM	MDR	3	0.5
CIP TCY IPM	MDR	1	0.2
AMC CTX CIP	MDR	2	0.4
CIP TCY SXT IPM	MDR	1	0.2
CIP NAL TCY IPM	MDR	1	0.2
CIP NAL TCY SXT	MDR	1	0.2
CIP GEN TCY IPM	MDR	1	0.2
CTX NAL TCY SXT	MDR	2	0.4
CTX CIP NAL IPM	MDR	2	0.4
AMP TCY SXT IPM	MDR	13	2.3
AMP GEN TCY SXT	MDR	1	0.2
AMP CIP SXT IPM	MDR	1	0.2
AMP CIP TCY SXT	MDR	7	1.2
AMP CHL SXT IPM	MDR	2	0.4
AMP CHL TCY IPM	MDR	1	0.2
AMP CHL TCY SXT	MDR	3	0.5
AMP CTX SXT IPM	MDR	2	0.4
AMP CTX TCY IPM	MDR	1	0.2
AMP CTX TCY SXT	MDR	33	5.8
AMP CTX GEN IPM	MDR	1	0.2
AMP CTX GEN TCY	MDR	1	0.2
AMP CTX CIP TCY	MDR	2	0.4
AMP CTX CIP GEN	MDR	1	0.2
AMP CTX CHL SXT	MDR	1	0.2
AMC CTX SXT IPM	MDR	1	0.2
AMC AMP SXT IPM	MDR	1	0.2
AMC AMP TCY IPM	MDR	1	0.2
AMC AMP TCY SXT	MDR	10	1.8
AMC AMP GEN TCY	MDR	1	0.2
AMC AMP CIP SXT	MDR	2	0.4
AMC AMP CHL SXT	MDR	1	0.2
AMC AMP CTX IPM	MDR	1	0.2
AMC AMP CTX SXT	MDR	6	1.1
AMC AMP CTX TCY	MDR	2	0.4
AMC AMP CTX CIP	MDR	2	0.4
CIP NAL TCY SXT IPM	MDR	1	0.2
CTX NAL TCY SXT IPM	MDR	1	0.2
CTX GEN TCY SXT IPM	MDR	1	0.2
CTX CIP GEN SXT IPM	MDR	1	0.2
CTX CIP GEN NAL TCY	MDR	1	0.2
CTX CHL CIP NAL IPM	MDR	1	0.2

AMP NAL TCY SXT IPM	MDR	2	0.4
AMP GEN TCY SXT IPM	MDR	3	0.5
AMP GEN NAL TCY SXT	MDR	2	0.4
AMP CIP TCY SXT IPM	MDR	1	0.2
AMP CIP NAL TCY IPM	MDR	1	0.2
AMP CIP NAL TCY SXT	MDR	4	0.7
AMP CHL NAL TCY SXT	MDR	5	0.9
AMP CTX TCY SXT IPM	MDR	2	0.4
AMP CTX NAL SXT IPM	MDR	1	0.2
AMP CTX NAL TCY SXT	MDR	10	1.8
AMP CTX CIP GEN SXT	MDR	1	0.2
AMP CTX CIP GEN NAL	MDR	1	0.2
AMP CTX CHL TCY SXT	MDR	3	0.5
AMP CTX CHL NAL SXT	MDR	2	0.4
AMP CTX CHL NAL TCY	MDR	1	0.2
AMC CTX CIP TCY IPM	MDR	1	0.2
AMC AMP TCY SXT IPM	MDR	3	0.5
AMC AMP NAL TCY SXT	MDR	7	1.2
AMC AMP CIP TCY SXT	MDR	3	0.5
AMC AMP CIP NAL TCY	MDR	1	0.2
AMC AMP CHL TCY SXT	MDR	4	0.7
AMC AMP CHL NAL SXT	MDR	1	0.2
AMC AMP CTX SXT IPM	MDR	4	0.7
AMC AMP CTX TCY SXT	MDR	17	3.0
AMC AMP CTX NAL IPM	MDR	1	0.2
AMC AMP CTX NAL SXT	MDR	2	0.4
AMC AMP CTX CIP SXT	MDR	1	0.2
AMC AMP CTX CIP NAL	MDR	1	0.2
CTX CIP GEN NAL TCY IPM	MDR	1	0.2
CTX CIP GEN NAL TCY SXT	MDR	1	0.2
AMP CIP NAL TCY SXT IPM	MDR	1	0.2
AMP CIP GEN NAL TCY SXT	MDR	4	0.7
AMP CHL GEN NAL SXT IPM	MDR	1	0.2
AMP CHL CIP NAL TCY SXT	MDR	1	0.2
AMP CTX NAL TCY SXT IPM	MDR	2	0.4
AMP CTX GEN NAL SXT IPM	MDR	1	0.2
AMP CTX CIP NAL TCY IPM	MDR	1	0.2
AMP CTX CIP NAL TCY SXT	MDR	5	0.9
AMP CTX CHL GEN NAL IPM	MDR	1	0.2
AMP CTX CHL CIP TCY SXT	MDR	1	0.2
AMC AMP CIP GEN NAL SXT	MDR	2	0.4
AMC AMP CHL TCY SXT IPM	MDR	1	0.2
AMC AMP CHL NAL TCY SXT	MDR	1	0.2
AMC AMP CHL CIP TCY SXT	MDR	2	0.4
AMC AMP CHL CIP NAL SXT	MDR	1	0.2
AMC AMP CTX TCY SXT IPM	MDR	4	0.7
AMC AMP CTX NAL SXT IPM	MDR	1	0.2

AMC AMP CTX NAL TCY SXT	MDR		3	0.5
AMC AMP CTX GEN SXT IPM	MDR		1	0.2
AMC AMP CTX GEN TCY SXT	MDR		1	0.2
AMC AMP CTX CIP TCY SXT	MDR		5	0.9
AMC AMP CTX CIP NAL IPM	MDR		1	0.2
AMC AMP CTX CIP NAL TCY	MDR		1	0.2
AMC AMP CTX CIP GEN TCY	MDR		1	0.2
AMC AMP CTX CIP GEN NAL	MDR		1	0.2
AMC AMP CTX CHL TCY IPM	MDR		1	0.2
AMC AMP CTX CHL TCY SXT	MDR		5	0.9
AMC AMP CTX CHL CIP NAL	MDR		2	0.4
AMP CTX CIP NAL TCY SXT IPM	MDR		3	0.5
AMP CTX CIP GEN NAL TCY SXT	MDR		5	0.9
AMP CTX CHL NAL TCY SXT IPM	MDR		2	0.4
AMP CTX CHL GEN NAL TCY SXT	MDR		1	0.2
AMP CTX CHL CIP NAL TCY SXT	MDR		3	0.5
AMC AMP CIP NAL TCY SXT IPM	MDR		1	0.2
AMC AMP CIP GEN NAL TCY SXT	MDR		1	0.2
AMC AMP CHL NAL TCY SXT IPM	MDR		3	0.5
AMC AMP CHL CIP NAL TCY SXT	MDR		1	0.2
AMC AMP CTX NAL TCY SXT IPM	MDR		2	0.4
AMC AMP CTX GEN NAL TCY IPM	MDR		1	0.2
AMC AMP CTX GEN NAL TCY SXT	MDR		2	0.4
AMC AMP CTX CIP NAL SXT IPM	MDR		1	0.2
AMC AMP CTX CIP NAL TCY SXT	MDR		7	1.2
AMC AMP CTX CHL NAL TCY SXT	MDR		2	0.4
AMC AMP CTX CHL CIP NAL SXT	MDR		1	0.2
CTX CIP GEN NAL TCY SXT IPM	MDR	Possible XDR	1	0.2
CTX CHL CIP GEN NAL TCY IPM	MDR	Possible XDR	1	0.2
AMP CHL CIP GEN TCY SXT IPM	MDR	Possible XDR	1	0.2
AMP CTX CIP GEN TCY SXT IPM	MDR	Possible XDR	1	0.2
AMP CTX CHL CIP GEN TCY SXT	MDR	Possible XDR	1	0.2
AMC AMP CHL CIP TCY SXT IPM	MDR	Possible XDR	1	0.2
AMC AMP CTX CIP TCY SXT IPM	MDR	Possible XDR	1	0.2
AMC AMP CTX CIP GEN TCY SXT	MDR	Possible XDR	1	0.2
AMC AMP CTX CHL GEN TCY SXT	MDR	Possible XDR	1	0.2
AMC AMP CTX CHL CIP TCY SXT	MDR	Possible XDR	1	0.2
AMP CTX CIP GEN NAL TCY SXT IPM	MDR	Possible XDR	1	0.2
AMC AMP CIP GEN NAL TCY SXT IPM	MDR	Possible XDR	1	0.2
AMC AMP CHL CIP NAL TCY SXT IPM	MDR	Possible XDR	1	0.2
AMC AMP CTX GEN NAL TCY SXT IPM	MDR	Possible XDR	1	0.2
AMC AMP CTX CIP NAL TCY SXT IPM	MDR	Possible XDR	6	1.1
AMC AMP CTX CIP GEN TCY SXT IPM	MDR	Possible XDR	2	0.4
AMC AMP CTX CIP GEN NAL SXT IPM	MDR	Possible XDR	1	0.2
AMC AMP CTX CIP GEN NAL TCY SXT	MDR	Possible XDR	9	1.6
AMC AMP CTX CHL NAL TCY SXT IPM	MDR	Possible XDR	2	0.4
AMC AMP CTX CHL CIP TCY SXT IPM	MDR	Possible XDR	1	0.2

AMC AMP CTX CHL CIP NAL SXT IPM	MDR	Possible XDR	1	0.2
AMC AMP CTX CHL CIP NAL TCY IPM	MDR	Possible XDR	1	0.2
AMC AMP CTX CHL CIP NAL TCY SXT	MDR	Possible XDR	2	0.4
AMC AMP CTX CHL CIP GEN TCY SXT	MDR	Possible XDR	2	0.4
AMC AMP CTX CHL CIP GEN NAL SXT	MDR	Possible XDR	1	0.2
AMC AMP CTX CHL CIP GEN NAL TCY	MDR	Possible XDR	1	0.2
AMP CTX CHL CIP GEN NAL TCY SXT IPM	MDR	Possible XDR	1	0.2
AMC AMP CTX CIP GEN NAL TCY SXT IPM	MDR	Possible XDR	3	0.5
AMC AMP CTX CHL CIP NAL TCY SXT IPM	MDR	Possible XDR	1	0.2
AMC AMP CTX CHL CIP GEN NAL SXT IPM	MDR	Possible XDR	1	0.2
AMC AMP CTX CHL CIP GEN NAL TCY SXT	MDR	Possible XDR	11	1.9
